# Hydroxychloroquine-induced pigmentation in rheumatic diseases: prevalence, clinical features and influencing factors

**DOI:** 10.1093/rheumatology/keae217

**Published:** 2024-04-08

**Authors:** Zi-Jing Yin, Pin Li, Juan Yu, Dachen Zuo, Hongtao Fan, Fayou Li, Juan Wang, Fei Gao, Weiqin Zhao, Shuya Wang, Sha Ma, Jing Wang

**Affiliations:** Department of Rheumatology, The First People's Hospital of Yunnan Province, The Affiliated Hospital of Kunming University of Science and Technology, Kunming, Yunnan, China; Department of Clinical Pharmacy, The First People's Hospital of Yunnan Province, The Affiliated Hospital of Kunming University of Science and Technology, Kunming, Yunnan, China; Department of Rheumatology, The First People's Hospital of Yunnan Province, The Affiliated Hospital of Kunming University of Science and Technology, Kunming, Yunnan, China; Department of Rheumatology, The First People's Hospital of Yunnan Province, The Affiliated Hospital of Kunming University of Science and Technology, Kunming, Yunnan, China; Department of Rheumatology, The First People's Hospital of Yunnan Province, The Affiliated Hospital of Kunming University of Science and Technology, Kunming, Yunnan, China; Department of Rheumatology, The First People's Hospital of Yunnan Province, The Affiliated Hospital of Kunming University of Science and Technology, Kunming, Yunnan, China; Department of Rheumatology, The First People's Hospital of Yunnan Province, The Affiliated Hospital of Kunming University of Science and Technology, Kunming, Yunnan, China; Department of Dermatology, The First People's Hospital of Yunnan Province, The Affiliated Hospital of Kunming University of Science and Technology, Kunming, Yunnan, China; Department of Rheumatology, The First People's Hospital of Yunnan Province, The Affiliated Hospital of Kunming University of Science and Technology, Kunming, Yunnan, China; Department of Rheumatology, The First People's Hospital of Yunnan Province, The Affiliated Hospital of Kunming University of Science and Technology, Kunming, Yunnan, China; Department of Rheumatology, The First People's Hospital of Yunnan Province, The Affiliated Hospital of Kunming University of Science and Technology, Kunming, Yunnan, China; Department of Rheumatology, The First People's Hospital of Yunnan Province, The Affiliated Hospital of Kunming University of Science and Technology, Kunming, Yunnan, China

**Keywords:** Pigmentation, HCQ, sun exposure, prevalence, clinical features, influencing factors

## Abstract

**Objective:**

To describe the clinical features of Chinese patients with HCQ-induced pigmentation and analyse the potential risk factors associated with HCQ-induced pigmentation.

**Methods:**

A cross-sectional study was conducted over a duration of 7 months, during which patients who had received HCQ treatment for >6 months were included. Data was collected through a structured questionnaire that encompassed demographic and geographic characteristics, information on HCQ and concomitant medication usage, sun exposure characteristics and hyperpigmentation-related characteristics. Univariate and multivariate analyses were employed to calculate the statistical association between HCQ-induced pigmentation and multiple variables.

**Results:**

Out of 316 patients, 83 (26.3%) patients presented hyperpigmentation during HCQ treatment. Hyperpigmentation was presented after a median duration of HCQ treatment of 12 months (interquartile range, 6.0–30.0 months) with a median cumulative dose of 108 g of HCQ (interquartile range, 36–288 g). The most frequently affected sites of pigmentation were the face (60.2%), lower limbs (36.1%) and hands (20.5%). There was a linear decrease in the incidence of pigmentation with increasing daily sun exposure time (*P* = 0.030). In the multivariate analysis, variables (cumulative HCQ dose and daily sun exposure time) were included in the final models. The results revealed an independent correlation between HCQ-induced pigmentation and daily sun exposure exceeding 1 h (OR: 0.431; 95% CI: 0.208–0.892; *P* = 0.023).

**Conclusions:**

The occurrence of HCQ-induced pigmentation is not uncommon, with an incidence rate of 26.3%. Daily sun exposure time exhibited a protective effect against HCQ-induced pigmentation.

Rheumatology key messages

- The occurrence of HCQ-induced pigmentation is not uncommon, with an incidence rate of 26.3%.
- Daily sun exposure time exhibited a protective effect against HCQ-induced pigmentation.
- Sun exposure was associated with HCQ-induced pigmentation while the causation and mechanism remain unclear.

## Introduction

Since the 1940s, chloroquine and HCQ have been utilized for the management of rheumatic diseases, including SLE, RA, dermatomyositis, cutaneous lupus, undifferentiated connective tissue disease, and seronegative inflammatory arthritis [1, 2]. HCQ is widely acknowledged to possess a favourable safety profile and exhibit cost-effectiveness. Nevertheless, the utilization of HCQ is constrained due to its diverse array of side effects. Retinopathy stands as the most frequently documented and researched adverse effect. Cutaneous side effects are often underestimated; however, they can significantly impede adherence to HCQ.

Cutaneous hyperpigmentation caused by antimalarials was first reported by Barr in 1944. During the Second World War, American soldiers in the South Pacific who were taking mepacrine for malaria prophylaxis presented with transverse blue pigmentation on their fingers and toenails [3]. Subsequent reports documented cases of hyperpigmentation occurring after treatment with all antimalarials, and a total of 29 articles reported a cumulative number of 116 patients who experienced hyperpigmentation following the administration of HCQ [4]. However, most of these cases focused on were Caucasians. To our knowledge, there was no comprehensive investigation into the clinical features of Chinese patients with HCQ-induced pigmentation. Only two studies investigated the risk factors associated with HCQ-induced pigmentation [5, 6]. The main identified risk factors include ecchymosis or bruising, antiplatelet agents, and oral anticoagulants. While some studies mentioned a potential relationship between sun exposure and HCQ-induced pigmentation [7–9], this aspect has not been thoroughly investigated.

The aim of this study was to describe the clinical features of Chinese patients with HCQ-induced pigmentation and analyse the correlation between sun exposure and HCQ-induced pigmentation.

## Methods

### Patients

This retrospective, monocentric cross-sectional study was conducted at The First People's Hospital of Yunnan Province, China over a 7-month period (May–November 2022) to investigate the use of HCQ in patients with systemic autoimmune disease. Patients who had received HCQ treatment for >6 months in the Department of Rheumatology and Immunology were included. A diagnosis of HCQ-induced pigmentation was made if typical pigmentation that could not be explained by toxins or other drugs, dermatoses, endocrinopathies, or nutritional conditions.

In all patients, data obtained through a structured questionnaire included demographic information, geographic characteristics, dosage and duration of HCQ treatment, concurrent medication use, presence of hyperpigmentation and characteristics of sun exposure. The distribution and clinical morphology of the skin lesions were assessed, accompanied by clinical photographs. An experienced dermatologist confirmed the diagnosis in all cases based on direct patient examination or review of patient images.

This study was approved by the Medical Ethics Committee and the Research Ethics Review Committee of the First People's Hospital of Yunnan Province (KHLL2022-KY041). Informed consent was obtained from all patients according to the Declaration of Helsinki.

### Annual sunshine duration and ultraviolet intensity

The data of annual sunshine duration and ultraviolet intensity (UVI) for the habitual residence of all patients were obtained from the ERA5 (ECMWF Re-Analysis 5) monthly averaged data on single levels from 1979 to the present. This was the fifth generation of reanalysis data of global climate and weather values over the past 40–70 years by the European Centre for Medium Range Weather Forecasts (ECMWF) [10]. We collected data from 2000 to 2022 to cover the time when all patients started using HCQ and averaged them.

### Adherence assessment

Patients were requested to assess the level of adherence using the MMAS-8 (Morisky Medication Adherence Scale) [11]. Highly adherent patients were identified with a score of 8 on the scale, medium adherers with a score of 6 to <8, and low adherers with a score of <6.

### Statistical analysis

The data were analysed using SPSS version 25.0 (SPSS Inc., Chicago, IL, USA). Continuous variables with normal distribution were presented as mean± standard deviation (SD); non-normal variables were reported as median (interquartile range). The median (minimum, maximum) was used to represent the data when the number of cases is less than or equal to 3. The means of two continuous, normally distributed variables were compared using an independent samples Student's test. The means of two continuous variables, which were not normally distributed, were compared using the MannWhitney *U* test. The frequencies of categorical variables were compared using Pearson χ2 or Fisher’s exact test, when appropriate. A test for trend was performed to ascertain the statistical significance of trends observed. The linear-by-linear association was used for trend in binomial proportions. Candidate variables that were considered clinically relevant or with a *P*-value <0.2 on univariate analysis were included in the multivariable model. The collinearity of variables was examined through multicollinearity diagnostics. A logistic regression test was performed for multivariate analysis to rule out possible confounding variables. Odds ratios (ORs) were calculated to assess the risk of each variable. Variables for inclusion were carefully chosen, given the number of events available, to ensure the parsimony of the final model. The final models were determined by a backward-LR variable selection approach in which the least significant variable was removed one by one after fitting a full model with the candidate variables. A *P*-value <0.05 was considered statistically significant.

## Results

### Baseline characteristics of the included population

A total of 316 patients who were treated with HCQ for over six months were enrolled. Among them, 214 (67.7%) patients had SLE, 50 (15.8%) patients had Sjogren’s syndrome (SS), 15 (4.7%) patients had RA, and 37 (11.7%) patients were classified as ‘other’. In addition to HCQ, 285 (90.2%) patients were treated with steroids, 213 (67.4%) patients with conventional synthetic disease-modifying anti-rheumatic drugs (csDMARDs), 6 (1.9%) patients with biological disease-modifying anti-rheumatic drugs (bDMARDs), 19 (6.0%) patients with csDMARDs combined with bDMARDs, and 4 (1.3%) patients with csDMARDs combined with targeted synthetic disease-modifying anti-rheumatic drugs (tsDMARDs). Fifteen (4.7%) patients were treated with Tripterygium Glycosides, a drug that was shown to have a risk of facial pigmentation. In patients with HCQ-induced pigmentation, 4 (4.8%) patients were treated with Tripterygium Glycosides while receiving HCQ. However, none of them presented with facial presentation. The hyperpigmentation was localized on the hands in three patients, on the foot in two and on the chest in one.

Of the 316 patients, we found 83 (26.3%) presented hyperpigmentation during HCQ treatment. Among them, 78 (94.0%) patients were female. The mean age was 40.8 ± 12.0 years. Hyperpigmentation was presented after a median duration of HCQ treatment of 12 months (interquartile range, 6.0–30.0 months) with a median cumulative dose of 108 g of HCQ (interquartile range, 36–288 g). The initial dose of HCQ administered to 214 (67.7%) patients exceeded the optimal dose recommended based on their body weight (5 mg/kg/day). The proportion of patients who received excessive HCQ treatment (initial dose/day>optimal dose/day) in patients with HCQ-induced pigmentation was 63.9%, which did not show a statistically significant difference compared with that in patients without HCQ-induced pigmentation (69.1%).

The comparison of baseline characteristics of patients with HCQ-induced pigmentation and patients without HCQ-induced pigmentation is summarized in Table 1. We did not find a significant difference between patients with HCQ-induced pigmentation and patients without HCQ-induced pigmentation in terms of age, sex, BMI, disease distribution, average patients’ adherence, previous ecchymosis or bruising, dosage and treatment duration of HCQ, and other drug intake. There was no linear trend between BMI and HCQ-induced pigmentation.

**Table 1.** Baseline characteristics of patients with HCQ-induced pigmentation compared with patients without HCQ-induced pigmentation

Characteristics	Patients with HCQ-induced pigmentation (n = 83)	Patients without HCQ-induced pigmentation (n = 233)	*P* value
**Patient characteristics**			
Age, mean (SD), years	40.8 (12.0)	42.6 (15.0)	0.249
Sex, female, *n* (%)	78.0 (94.0)	207.0 (88.8)	0.177*
BMI, mean (SD), kg/m^2^	22.9 (3.4)	22.2 (3.4)	0.521
Body mass index by category, *n* (%)			0.683^a^
Underweight (<18.5) (*n* = 23)	9 (10.8)	24 (10.3)	
Normal (≥18.5, <25) (*n* = 157)	60 (72.3)	168 (72.1)	
Overweight (≥25, <30) (*n* = 34)	13 (15.7)	34 (14.6)	
Obese (≥40) (*n* = 5)	1 (1.2)	7 (3.0)	
**Disease distribution**			
SLE, *n* (%)	58 (69.9)	156 (67.0)	0.642
SS, *n* (%)	14 (16.9)	36 (15.5)	0.761
RA, *n* (%)	3 (3.6)	12 (5.2)	0.572
Other, *n* (%)	8 (9.6)	29 (12.4)	0.495
**Medication characteristics**			
Ever used steroids, *n* (%)	75 (90.4)	210 (90.1)	0.951
Ever used csDMARDs, *n* (%)	65 (78.3)	171 (73.4)	0.376
Ever used tripterygium glycosides, *n* (%)	4 (4.8)	11 (4.7)	1.000
Ever used bDMARDs, *n* (%)	9 (10.8)	16 (6.9)	0.249
Ever used tsDMARDs, *n* (%)	3 (3.6)	1 (0.4)	0.097*
**Characteristics of HCQ use**			
Duration of HCQ use, median (interquartile range), m	52.8 (27.5, 90.9)	38.5 (20.1, 84.7)	0.150*
Cumulative HCQ dose, median (interquartile range), g	472.4 (181.8, 864.0)	384.0 (181.4, 736.5)	0.200
Initial dose/day *vs* Optimal dose/day^b^			
Initial dose/day>Optimal dose/day	53 (63.9)	161 (69.1)	0.770
Initial dose/day ≤Optimal dose/day	30 (36.1)	72 (30.9)	
Average patients’ adherence for HCQ, median (interquartile range)	6.0 (5.0, 7.0)	6.8 (5.0, 7.0)	0.362
Average patients’ adherence for HCQ by category, *n* (%)			0.539^a^
Highly adherers, (*n* = 26)	9 (10.8)	25 (10.7)	
Medium adherers, (*n* = 103)	42 (50.6)	130 (55.8)	
Low adherers, (*n* = 90)	32 (38.6)	78 (33.5)	
**Reported risk factors for HCQ side effects**			
Ever used Antiplatelet agents, *n* (%)	22 (26.5)	59 (25.3)	0.832
Ever used Anticoagulants, *n* (%)	4 (4.8)	13 (5.6)	1.000
Secondary to ecchymosis or bruising, *n* (%)	8 (9.6)	—	

a Linear-by-linear association.

b Optimal dosage determined by individual body weight (5 mg/kg/day).

* *P*-value<0.2.

BMI: Body mass index; HCQ: hydroxychloroquine; csDMARDs: conventional synthetic disease-modifying anti-rheumatic drugs; bDMARDs: biological disease-modifying anti-rheumatic drugs; tsDMARDs: targeted synthetic disease-modifying anti-rheumatic drugs.

### Characteristics of patients with HCQ-Induced pigmentation

**Figure 1.**
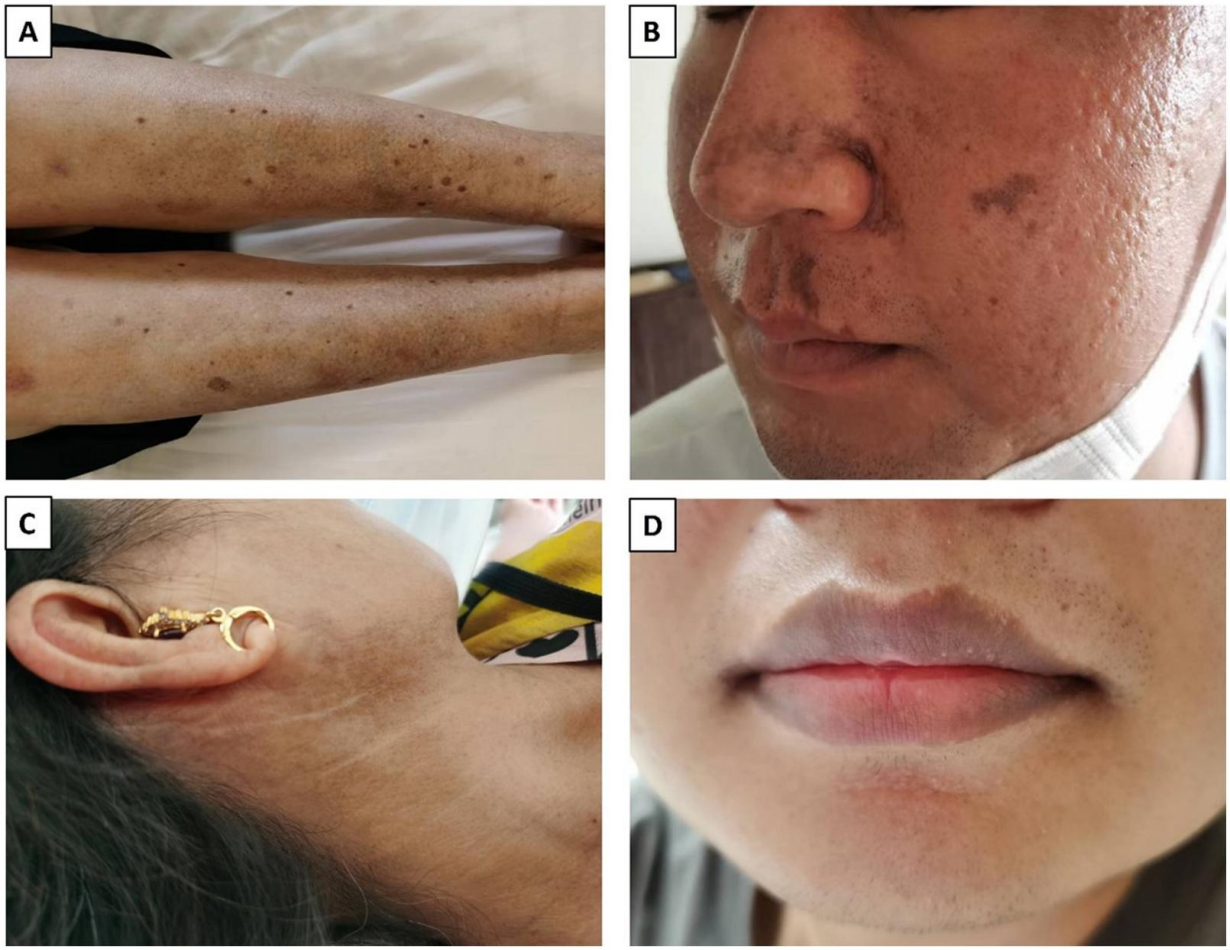
Patient photographs. (A) Reticulated macular gray pigmentation involving the anterior aspect of the shins. (B) Pigmentation occurs at the site of the rash, secondary to herpes zoster infection. (C) Pigmentation localized to the cervical region. (D) Pigmentation localized to the vermilion zone of the lips

Pigmented lesions in the study were observed to be brown to black. Hyperpigmentation was found to be localized to the facial region in 50 (60.2%) patients, to the periorbital skin in 6 (7.2%), to the lips in 4 (4.8%), to the cervical region in 5 (6.0%), to the anterior chest in 2 (2.4%), to the upper limb in 13 (15.7%), to the hands in 17 (20.5%), to the Nail in 1 (1.2%), to the dorsum in 1 (1.2%), to the lower limbs in 30 (36.1%), to the feet in 6 (7.2%), to the whole body in 5 (6.0%) (Table 2). Fifty-two (62.7%) patients reported that the pigmentation appears uniformly, that is, the entire skin of lesions appears pigmented; 29 (34.9%) patients reported spotty or flaky pigmentation; 2 (2.4%) patients reported pigmentation occurs at the site of the rash, one secondary to herpes zoster (Figure 1B) and one secondary to urticaria vasculitis.

**Table 2.** The site of skin pigmentation of patients with HCQ-induced pigmentation

Sites of skin pigmentation	Patients with HCQ-induced pigmentation (*n* = 83)	Duration of HCQ use, median (interquartile range), m	Cumulative HCQ dose, median (interquartile range), g
Facial region, *n* (%)	50 (60.2)	12.0 (5.5, 24.0)	108.0 (36.0, 288.0)
Periorbital skin, *n* (%)	6 (7.2)	9.0 (2.8, 36.0)	99.0 (33.0, 378.0)
Lip, *n* (%)	4 (4.8)	6.0 (6.0, 6.0)	54.0 (45.1, 67.5)
Cervical region, *n* (%)	5 (6.0)	6.0 (3.3,30.0)	36.0 (19.5, 306.0)
Anterior chest, *n* (%)	2 (2.4)	1.0–4.5	12.0–30.0
Upper limb, *n* (%)	13 (15.7)	12.0 (6.0, 48.0)	72.0 (51.0, 486.0)
Hands, *n* (%)	17 (20.5)	18.0 (7.0, 27.0)	216.0 (63.0, 288.0)
Nail, *n* (%)	1 (1.2)	168.0	1512.0
Dorsum, *n* (%)	1 (1.2)	36.0	216.0
Lower limbs, *n* (%)	30 (36.1)	12.0 (5.5, 36.0)	81.0 (45.0, 351.0)
Feet, *n* (%)	6 (7.2)	15.0 (11.0, 46.5)	180.0 (66.0, 490.5)
The whole body, *n* (%)	5 (6.0)	6.0 (4.0, 52.0)	48.0 (21.0, 180.0)

Further analysis of the correlation between pigmentation sites and the duration of HCQ use as well as cumulative dosage revealed that cases involving pigmentation on the anterior chest, cervical region, and lips exhibited shorter durations and lower cumulative doses of HCQ. The case involving pigmentation on the nail, despite being limited to only one patient, exhibited the longest duration of HCQ use (168 months) and the highest cumulative dose (1512 g).

At the onset of pigmented lesions, 8 (9.6%) patients reported a prior occurrence of ecchymosis or bruising, while 22 (26.5%) patients were administered antiplatelet agents and 4 (4.8%) patients received anticoagulants. These factors were identified as reported risk factors associated with HCQ-induced hyperpigmentation.

Seven (8.4%) patients reported HCQ discontinuation due to hyperpigmentation, and 14 (16.9%) patients reported spontaneous complete or partial regression of skin pigmentation after withdrawal of HCQ.

Since some patients developed pigmentation shortly after HCQ administration, we compared the characteristics of patients who experienced HCQ-induced pigmentation within 12 months of treatment (SDC patients) with those who had it after >12 months (LDC patients) (Table 3). We observed that BMI was significantly lower in SDC patients (20.9 ± 2.8 kg/m^2^) compared with LDC patients (22.7 ± 3.7 kg/m^2^) (*P* = 0.019). The proportion of patients who received excessive HCQ treatment (initial dose/day > optimal dose/day) in SDC patients was 62.2%, which did not demonstrate a statistically significant difference compared with that in LDC patients (65.2%).

**Table 3.** Comparison of patients with hyperpigmentation within 12 months with those experiencing hyperpigmentation after 12 months

Characteristics	SDC-Patients^a^(*n* = 37)	LDC-Patients^b^(*n* = 46)	*P* value
Age, mean (SD), y	40.0 (10.8)	41.4 (12.9)	0.600
Sex, female, *n* (%)	35 (94.6)	43 (93.5)	1.000
BMI, mean (SD), kg/m^2^	20.9 (2.8)	22.7 (3.7)	0.019**
Initial dose of HCQ			
0.1 g/day	—	1 (2.2)	
0.2 g/day	12 (32.4)	6 (13.0)	
0.3 g/day	—	1 (2.2)	
0.4 g/day	25 (67.6)	38 (82.6)	
Initial dose/day *vs* Optimal dose/day ^d^			
Initial dose/day>Optimal dose/day	23 (62.2)	30 (65.2)	0.083
Initial dose/day ≤ Optimal dose/day	14 (37.8)	16 (34.8)	
Annual sunshine duration at habitual residence, median (interquartile range), h	1660.9 (1617.2,1691.5)	1660.1 (1586.1,1686.6)	0.185
UVI at habitual residence, median (interquartile range), MJ/m^2^	691.7 (672.0,705.1)	688.9 (661.8,700.4)	0.399
Daily work style, *n* (%)			
Indoor	34 (91.9)	44 (95.7)	0.801
Outdoor	3 (8.1)	2 (4.3)	
Daily sun exposure time			0.543^c^
<half-hour	16 (43.2)	16 (34.8)	
≥half-hour, <1 h	15 (40.5)	22 (47.8)	
≥1 h	6 (16.2)	8 (17.4)	
Ever used sun protection measures, *n* (%)	28 (75.7)	38 (82.6)	0.437
Frequency of sun protection, *n* (%)			0.571^c^
Never	9 (24.3)	8 (17.4)	
<3.5 days/week	1 (2.7)	5 (10.9)	
>3.5 days/week	18 (48.6)	30 (65.2)	
Everyday	9 (24.3)	3 (6.5)	

a SDC (short disease course)-Patients, patients with HCQ-induced pigmentation occurs <12 months after HCQ treatment.

b LDC (long disease course)-Patients, patients with HCQ-induced pigmentation occurs >12 months after HCQ treatment.

c Linear-by-linear association.

d Optimal dosage determined by individual body weight (5 mg/kg/day).

* *P*-value<0.2.

** *P*-value<0.05.

Although there was no significant difference in sun exposure characteristics between SDC patients and LDC patients, it appeared that LDC patients tend to exhibit a greater emphasis on sun protection. The percentage of LDC patients who took sun protection measures during HCQ use was 82.6%, while for SDC patients, it was 75.7%. However, this disparity did not reach statistical significance.

### The comparison of sun exposure characteristics between patients with HCQ-induced pigmentation and patients without HCQ-induced pigmentation

The comparison of sun exposure characteristics between patients with HCQ-induced pigmentation and patients without HCQ-induced pigmentation is summarized in Table 4. We did not observe a significant difference in annual sunshine duration and UVI of the habitual residence between patients with HCQ-induced pigmentation and those without. Furthermore, no significant difference was observed between patients with HCQ-induced pigmentation and those without in terms of their daily work style, adherence to sun protection measures, and frequency of sun protection. There was no significant linear trend observed between annual sunshine duration or UVI and the pigmentation induced by HCQ. Surprisingly, we observed a linear decrease in the occurrence of pigmentation with the daily sun exposure time increased (*P* = 0.030).

**Table 4.** Comparison of sun exposure characteristics between patients with HCQ-induced pigmentation and those without HCQ-induced pigmentation.

Characteristic of sun exposure	Patients with HCQ-induced pigmentation (n = 83)	Patients without HCQ-induced pigmentation (n = 233)	*P* value
Annual sunshine duration at habitual residence, median (interquartile range), h	1660.9 (1597.3, 1686.6)	1658.0 (1574.1, 1686.6)	0.196*
Annual sunshine duration at habitual residence by category, *n* (%)			0.402^a^
<1400	4 (4.8)	13 (5.6)	
1400–1500	1 (1.2)	7 (3.0)	
1500–1600	16 (19.3)	43 (18.5)	
1600–1700	52 (62.7)	152 (65.2)	
≥1700	10 (12.0)	18 (7.7)	
UVI at habitual residence, median (interquartile range), MJ/m^2^	691.7 (671.8, 701.3)	686.1 (659.5,700.4)	0.234
UVI at habitual residence by category, *n* (%)			0.704^a^
<500	1 (1.2)	5 (2.1)	
500–600	3 (3.6)	8 (3.4)	
600–700	57 (67.8)	160 (68.7)	
≥700	22 (26.5)	60 (25.8)	
Daily work style, *n* (%)			0.177*
Indoor	78 (94.0)	207 (88.8)	
Outdoor	5 (6.0)	26 (11.2)	
Daily sun exposure time			0.030^**^^a^
<half-hour	32 (38.6)	64 (27.5)	
≥half-hour, <1 h	37 (44.6)	108 (46.4)	
≥1 h	14 (16.9)	61 (26.2)	
Ever used sun protection measures, *n* (%)	66 (79.5)	197 (84.5)	0.292
Sun protection measures, *n* (%)			0.197^a^
Sunscreen	0 (0.0)	1 (0.4)	
Sunshade	1 (1.2)	6 (2.6)	
Sun-protective clothing	2 (2.4)	12 (5.2)	
Avoid going out	1 (1.2)	3 (1.3)	
Use multiple measures	62 (74.7)	175 (75.1)	
Frequency of sun protection, *n* (%)			0.831^a^
never	17 (20.5)	36 (15.5)	
<3.5 days/week	6 (7.2)	24 (10.3)	
>3.5 days/week	48 (57.8)	161 (69.1)	
Everyday	12 (14.5)	12 (5.2)	

a Linear-by-linear association.

* *P*-value<0.2.

** *P*-value<0.05.

MJ/m2: megajoule per square metre.

### Multivariable analysis for the risk of HCQ-induced pigmentation

The candidate variables (sex, age, BMI, duration of HCQ use, cumulative HCQ dose, annual sunshine duration at habitual residence, UVI at habitual residence and daily sun exposure time) that were considered clinically relevant or had a *P*-value <0.2 on univariate analysis were included in the multivariable model. Due to the collinearity between annual sunshine duration and UVI, the variable with a *P*-value <0.2 in the univariate analysis, which is UVI, was selected for inclusion in the model. The final models were determined using a backward-LR variable selection approach. Variables (sex, age, BMI, duration of HCQ use, and UVI at habitual residence) with a probability greater than the removal value of *P* = 0.20 were removed. Variables (cumulative HCQ dose and daily sun exposure time) were included in the final models (Table 5). We found that HCQ-induced pigmentation was independently associated with the daily sun exposure time for >1 h (OR: 0.431; 95% CI: 0.208–0.892; *P* = 0.023).

**Table 5.** Multivariable Analysis for the risk of HCQ-induced pigmentation

	OR	95% CI	*P* value
Cumulative HCQ dose, g	1.000	1.000–1.001	0.112
Daily sun exposure time			
<half-hour			0.069
≥half-hour, <1 h	0.659	0.372–1.165	0.152
≥1 h	0.431	0.208–0.892	0.023

In the multivariable model, candidate variables for inclusion included sex, age, BMI, duration of HCQ use, cumulative HCQ dose, annual sunshine duration at habitual residence, UVI at habitual residence and daily sun exposure time (<half-hour;≥half-hour,<1 h;≥1 h).

CI: confidence interval; OR: odds ratio.

## Discussion

In this study, we included 316 patients who had received HCQ treatment for over 6 months. We observed that 83 (26.3%) patients presented hyperpigmentation during HCQ treatment, with the majority being female (94.0%). These results were in line with previous research indicating that the incidence of cutaneous hyperpigmentation varies from 7% to 29%, with the majority of patients being female (>90%) [5, 6, 12]. However, this does not imply that women are inherently more prone to hyperpigmentation. Given that 83.5% of the patients enrolled in our study had SLE or SS, two conditions with a higher prevalence among females, it is more likely that the occurrence of hyperpigmentation in female patients is related to their diagnosis rather than being solely attributed to hyperpigmentation itself. In our study, only 106 (33.5%) patients were aged above 50 years, which was lower than previously reported (>80%) [4, 13]. However, the mean age of patients with HCQ-induced pigmentation (40.8 years) was basically consistent with those of previous studies (34.3–44.7 years) [5, 6, 12]. This discrepancy may be attributed to the fact that most respondents willing to participate and complete our questionnaire survey were under the age of 50.

Given that a higher proportion of patients with SLE received treatment with HCQ compared with patients with SS, RA, and other rheumatic diseases, the incidence of cutaneous hyperpigmentation was found to be highest in the SLE group. In our study, the occurrence of cutaneous hyperpigmentation ranked in descending order as follows: SLE (67.7%), SS (15.8%), other rheumatic diseases (11.7%) and RA (4.7%).

Cutaneous hyperpigmentation reactions are described as yellow-brown to slate grey or black pigmentation. These changes are observed on nearly all locations of the body, including hairline, lips, face, torso (upper back, shoulders and upper chest), extremities (forearms, hands, thigh, pretibial, feet and nail beds) and oral mucosa (hard palace and gingivae) [4, 8, 13]. Our findings were consistent with this description. In our study, the most frequently affected sites of pigmentation were the face (60.2%), lower limbs (36.1%), and hands (20.5%), all of which are commonly exposed to sunlight. We also observed different forms of pigmentation in our study. Some patients presented uniformly pigmentation, some presented spotty or flaky pigmentation, and some presented pigmentation at the site of the rash or lesions. Pigmentation within lesions was also reported by Reynaert *et al.* where the patient presented with similar pigmentation within atrophic scars of discoid lupus erythematosus after being treated with mepacrine [14]. Furthermore, specific types of hyperpigmentation were documented in the literature as well. Coulombe *et al.* [15] reported a 48-year-old man presenting with a three-month history of mottled blue-grey hyperpigmentation on his face and areas of previous bruising on his arms and legs. Lau *et al.* [16] reported a 53-year-old male having a peculiar pattern of skin pigmentation that exclusively affected the superficial venous network in both lower extremities (serpentine supravenous hyperpigmentation, SSH), which is postulated to develop upon extravasation of a noxious agent from the superficial veins into the epidermal skin layer after venous endothelial damage, leading to disruption of melanogenesis and eventual skin hyperpigmentation.

Previous studies demonstrated that the time interval until the manifestation of hyperpigmentation caused by antimalarials varies between 4 and 70 months [17]. Regarding HCQ, Jallouli *et al.* [5] reported that cutaneous hyperpigmentation appeared after a median duration of HCQ treatment of 73.2 months (range, 3–264 months), while Bahloul *et al.* [6] reported a median duration of 32 months (range: 6–108 months). Our study found that hyperpigmentation occurred after a median duration of HCQ treatment of 12 months (range, 4.5 months–28.5 months), which was shorter than in previous studies. Additionally, the cumulative doses of HCQ taken by patients at the onset of pigmentation in our study [126 g (range, 36–288 g)] were comparatively lower than those reported in the studies conducted by Jallouli *et al.* [5] [720 g (range, 36–3168 g)] and Bahloul *et al*. [6] [361 g (range, 36–1314 g)]. Our study, employing a design similar to that of Bahloul *et al.* [6], aimed to investigate the incidence of hyperpigmentation and characteristics of patients who developed it by selecting individuals treated with HCQ for >6 months. However, Bahloul *et al.*‘s study only included 41 patients, out of whom only 12 exhibited hyperpigmentation. This limited sample size may have introduced bias into the results. In Jallouli *et al.*’s case–control study [5], 24 patients with hyperpigmentation were specifically chosen while controls consisted of 517 patients from the PLUS study. Nevertheless, there might be selection bias in choosing these particular cases displaying typical hyperpigmentation. It is worth noting that these patients may have used HCQ for an extended duration and accumulated higher cumulative doses compared with other cases. Compared with Jallouli *et al.*‘s data, the data of our study aligned more closely with Bahloul’s due to its similar design; however, our sample size was larger. Due to the above differences, additional multicentre studies with large samples are needed. Furthermore, we compared the initial dose of HCQ between SDC patients and LDC patients; however, no significant difference was observed.

It remains uncertain whether there is a time-dependent or dose-dependent correlation between hyperpigmentation and HCQ. Puri *et al.* proposed that the pigmentation is dependent on dosage, as mucosal hyperpigmentation occurs after a cumulative dose exceeding 400 g. They observed a gradual reduction in pigmentation over several months following discontinuation of the drug, indicating a dose-dependent relationship [18]. However, this conclusion was drawn from case reports involving only two patients. The findings of our study indicated that the duration and cumulative dosage of HCQ varied depending on the location of pigmentation. Our study revealed that cases involving pigmentation on the anterior chest, cervical region, lips, facial region, and upper limb exhibited shorter durations and lower cumulative doses of HCQ. These sites are more susceptible to sun exposure. In our study, patients also reported complete or partial regression of pigmentation following discontinuation of the drug. However, it should be noted that this does not imply a dose-dependent relationship with pigmentation. In our multivariate regression analysis, duration of HCQ use and cumulative HCQ dose were ultimately not included in the final model. Therefore, consistent with the conclusion drawn from a recent case–control study [5], we did not observe a significant association between the duration and cumulative dose of HCQ and the occurrence of hyperpigmentation. These results suggest that, unlike with HCQ retinopathy [1], HCQ treatment duration and cumulative HCQ dose might not major risk factors for pigmented lesions. Nevertheless, due to limitations inherent in our cross-sectional study design as well as in the case–control study conducted by Jallouli *et al.* [5], future research with improved study designs is warranted to confirm this association.

The optimal dose of HCQ is based on body weight and should not exceed 5 mg/kg/day in order to minimize toxicity, including retinal damage [19, 20]. However, approximately two-thirds of the patients (67.7%) in our study initially received a higher than optimal dose of HCQ. This discrepancy arises from the fact that the optimal dose is established with consideration for the risk of retinal toxicity rather than maximizing HCQ efficacy. Nevertheless, it has been demonstrated that an effective dose for HCQ is 6.5 mg/kg/day [19]. Therefore, in clinical practice, most rheumatologists tend to initiate treatment with a fixed dose of HCQ (400 mg/day) and subsequently reduce it to 200 mg/day according to the efficacy. Our study demonstrated that SDC patients had a significantly lower BMI compared with LDC patients. However, there was no significant difference in the proportion of patients whose daily initial HCQ dose exceeded the optimal dose between SDC patients and LDC patients. The rate of initial HCQ overuse (initial dose/day > optimal dose/day) in patients who developed pigmentation did not differ significantly from that in patients who did not develop pigmentation. There was no linear trend between BMI and HCQ-induced pigmentation. Therefore, although most patients in our study received an initial HCQ dose exceeding the weight-based optimal dose, our findings do not suggest a correlation between excessive use of HCQ and hyperpigmentation.

The mechanism underlying hyperpigmentation remains poorly understood. Histology typically reveals the presence of brown-pigmented granules of iron [21], melanin [13, 18], or both [5, 9, 22] deposited in the basal epidermis. Early studies suggested that hemosiderin is responsible for hyperpigmentation [21]. Antimalarials were previously primarily used against inflammation and hemosiderin resulting from inflammation-induced bleeding contributes to hyperpigmentation [22, 23]. However, it is currently believed that increased production of melanin is the primary mechanism involved [17]. Some studies have shown that chloroquine binds to melanin and histologically detectable granules consist of a melanin-chloroquine complex, suggesting that antimalarials like chloroquine and HCQ directly stimulate the melanocytes [23, 24]. Additionally, antimalarial drugs were found to stimulate melanocytes either directly or by elevating levels of androgens which in turn stimulate melanocytes [22]. Histology shown that an increased superficial dermal deposition of iron in the hyperpigmented skin compared with normal skin, which suggests that the induction of melanocytes activation may due to high hemosiderin content [4, 5, 25].

It is also not clear why HCQ treatment discontinuation can ameliorate hyperpigmentation. Histological studies demonstrated that HCQ-induced pigmentation could occur in the epidermis, dermis and hypodermis [5]. As previously mentioned, HCQ can stimulate melanocytes to produce melanin [23, 24]. Discontinuation of HCQ leads to a reduction and gradual elimination of this induction factor. The epidermal layer of the human body is known to undergo renew approximately every 28 days, while the dermis layer has a longer metabolic time for renewal. Therefore, with the elimination of HCQ as an induction factor and natural skin metabolism renewal, pigmentation may diminish or disappear over time. However, further research is necessary to confirm the exact mechanism.

Risk factors that have been reported so far included bruising, corticosteroid use, oral anticoagulants, antiplatelet agents, antiphospholipid syndrome and skin trauma. However, the findings of our study do not support previous research. In our study, we found that HCQ-induced pigmentation was independently associated with the daily sun exposure time for >1 h. The daily sun exposure time exhibited a protective effect against HCQ-induced pigmentation (OR = 0.431 < 1), and there was a linear decrease in the incidence of pigmentation with increasing daily sun exposure time. This contradicts our previous understanding. It is well known that ultraviolet radiation is one of the most well characterized factors governing skin pigmentation. Exposure to ultraviolet radiation results in synthesis of melanin pigments within highly specialized cells termed melanocytes [26]. As mentioned above, histopathological findings shown that melanin is responsible for hyperpigmentation [13, 18]. Previous case reports indicated an association between sun exposure and HCQ-induced pigmentation. Millard *et al.* [7] reported a 48-year-old female patient who was advised about general photoprotection during treatment with HCQ. After 1 year, the patient presented striking slate grey macular pigmentation affecting her face, neck, trunk, axillae and posterior thighs, thereby involving both sun-exposed and sun-protected sites. However, her hyperpigmentation improved in the winter months.

The association of reduced pigmentation with higher sun exposure was unexpected and remains unexplained. Longer sunshine duration and higher UVI may lead to more use of sun protection measures, particularly for patients with SLE, as excessive sun exposure can exacerbate melanin production and trigger disease onset. In our study, there was no significant difference in the use of sun protection measures between patients who developed pigmentation and those who did not. Most patients preferred employing multiple concurrent sun protection measures such as sunshade, sunscreen, and sun-protective clothing. Our current study findings only suggest a correlation between reduced pigmentation and higher sun exposure; however, the causation and underlying interaction mechanism remain unclear. Further validation studies are needed.

The limitations of our study primarily stem from its monocentric nature. Ninety-two percent of the patients included in our study were from Yunnan Province, China, with small variation in latitude. However, due to the unique geographical characteristics of Yunnan Province, there is a significant disparity in altitude among different regions, resulting in substantial differences in sunshine duration and ultraviolet radiation intensity. This aspect partially compensates for the aforementioned limitation in our study design. Furthermore, although pigmentation influenced patient compliance with HCQ due to aesthetic concerns, participants enrolled in our study were unwilling to undergo a skin biopsy as it did not impact their disease diagnosis and treatment. Consequently, histopathological evidence is lacking within our study findings. To address this deficiency, an experienced dermatologist confirmed the diagnosis for all cases.

## Conclusion

The occurrence of HCQ-induced pigmentation is not uncommon, with an incidence rate of 26.3%. Daily sun exposure time exhibited a protective effect against HCQ-induced pigmentation. The causation and underlying interaction mechanism of the association between decreased pigmentation and increased sun exposure remain unclear.

## Data Availability

The data underlying this article are available in the article.
